# Ca^2+^-Mediated Signaling Pathways: A Promising Target for the Successful Generation of Mature and Functional Stem Cell-Derived Pancreatic Beta Cells In Vitro

**DOI:** 10.3390/biomedicines11061577

**Published:** 2023-05-29

**Authors:** Razik Bin Abdul Mu-u-min, Abdoulaye Diane, Asma Allouch, Heba H. Al-Siddiqi

**Affiliations:** Diabetes Research Center, Qatar Biomedical Research Institute (QBRI), Hamad Bin Khalifa University (HBKU), Qatar Foundation (QF), Doha P.O. Box 34110, Qatar; adiane@hbku.edu.qa (A.D.);

**Keywords:** beta cells, calcium signaling, diabetes, stem cells, differentiation

## Abstract

Diabetes mellitus is a chronic disease affecting over 500 million adults globally and is mainly categorized as type 1 diabetes mellitus (T1DM), where pancreatic beta cells are destroyed, and type 2 diabetes mellitus (T2DM), characterized by beta cell dysfunction. This review highlights the importance of the divalent cation calcium (Ca^2+^) and its associated signaling pathways in the proper functioning of beta cells and underlines the effects of Ca^2+^ dysfunction on beta cell function and its implications for the onset of diabetes. Great interest and promise are held by human pluripotent stem cell (hPSC) technology to generate functional pancreatic beta cells from diabetic patient-derived stem cells to replace the dysfunctional cells, thereby compensating for insulin deficiency and reducing the comorbidities of the disease and its associated financial and social burden on the patient and society. Beta-like cells generated by most current differentiation protocols have blunted functionality compared to their adult human counterparts. The Ca^2+^ dynamics in stem cell-derived beta-like cells and adult beta cells are summarized in this review, revealing the importance of proper Ca^2+^ homeostasis in beta-cell function. Consequently, the importance of targeting Ca^2+^ function in differentiation protocols is suggested to improve current strategies to use hPSCs to generate mature and functional beta-like cells with a comparable glucose-stimulated insulin secretion (GSIS) profile to adult beta cells.

## 1. Introduction

Diabetes mellitus, commonly known as diabetes, is a collection of chronic and metabolic disorders typically observed with heightened blood glucose levels over an extended period. Insulin deficiency leading to impaired glucose metabolism is widely deemed to be the mechanism for the onset and effect of the disease [1,2]. The International Diabetes Federation (IDF) has been assessing the incidence of diabetes globally. Recent statistics have revealed that in 2021, 537 million adults live with diabetes, and the numbers are expected to increase to 783 million by 2045. Additionally, 6.7 million deaths were recorded due to the comorbidities of diabetes (www.idf.org, (accessed on 16 November 2022)). The American Diabetes Association (ADA) has classified diabetes into primarily two categories: type 1 diabetes (T1DM) and type 2 diabetes (T2DM) [3]. T1DM is a result of selective immune-mediated pancreatic beta-cell destruction, leading to a nearly complete deficiency of insulin production in the body. Although only 5–10% of all cases of diabetes are T1DM, it encompasses 80–90% of diabetes in children and adolescents [4,5,6]. T2DM, on the other hand, occurs when individuals have insulin resistance and a relative insulin deficiency due to beta cell dysfunction [2]. T2DM accounts for 90–95% of all cases of diabetes. Other types of diabetes include gestational diabetes mellitus (GDM), neonatal diabetes, and maturity-onset diabetes of the young (MODY) [7]. It is now well known that all forms of diabetes commonly share a dysfunction of the pancreatic beta cells that negatively impacts insulin secretion [4]. Unhealthy lifestyles, increasing global obesity in children and young adults, and an aging population are some of the risk factors for the increasing diabetes population of the world [2,8]. Diabetes is also associated with additional health burden through various macrovascular (stroke, peripheral artery disease, coronary heart diseases, and myocardial infarctions) and microvascular (neuropathy, retinopathy, and nephropathy) complications, which lead to diminished quality of life and premature death [2]. These diabetes-coupled complications also pose an immense financial, social, and medical challenge to both western and developing countries. The global healthcare expenses for managing diabetes were estimated to be USD 850 billion in 2017 [9,10,11,12,13].

T1DM treatment currently involves controlling glycemia through daily insulin supplementation through insulin injections or insulin pumps with integrated glucose monitors [14,15]. Although lifesaving, these invasive treatment strategies remain imperfect, as they could result in acute hypoglycemia and lead to heart and kidney failure. Cadaveric islet transplantation using the Edmonton protocol has been demonstrated as an ideal and effective treatment for T1DM, which could allow temporal exogenous insulin independence [16]. This treatment option is increasingly unrealistic for the growing number of patients with T1DM due to its high costs, lack of sufficient donors, and potential risk of tissue rejection resulting in lifelong immunosuppressive drugs [17,18]. Current T2DM treatment involves a combination of lifestyle and pharmacological interventions. Evidence suggests that regulated pharmacological interventions, along with dietary intake, physical exercise, and adequate sleep, are important to prevent aggravation of insulin resistance [19,20,21,22,23].

Recent progress in the field of regenerative medicine has focused on the generation of surrogate and transplantable functional pancreatic beta cells from human pluripotent stem cells (hPSCs) to treat diabetes. Patient-specific pluripotent stem cell (PSC)-derived beta cells, also known as autologous PSC-derived beta cells, would help circumvent the inadequate islet supply and/or allogeneic immune rejection, thereby being an unlimited source of beta cells for diabetes therapy. More importantly, patient-specific stem cell-derived beta cells can also be used in vitro to study diabetes-related mutations such as inherited monogenic diabetes as well as disease progression [24,25]. To that end, massive efforts are undertaken to differentiate hPSCs efficiently and reproducibly into insulin-expressing beta cells using multi-stage directed differentiation protocols that simulate the specific stages of pancreas development. However, differentiation attempts do not always result in functional insulin-expressing beta cells, and instead, the differentiated beta cells show an immature phenotype with impaired response to glucose-stimulated insulin secretion (GSIS) [18,26]. Both in vivo and in vitro studies consistently demonstrated that insulin release from electrically excitable pancreatic beta cells is a calcium (Ca^2+^)-dependent process. Therefore, defects in calcium signaling could be one of the underlying reasons for the immature and non-functional phenotypes observed in differentiated hPSC-derived pancreatic beta cells in vitro. In this review, we first describe the role of calcium signaling in pancreatic beta cell function and secondly highlight and summarize recent progress on the implications of impaired calcium signaling in defective insulin secretion in hPSC-derived β cells, and consequently, how targeting calcium signaling might be a new and interesting strategy to improve in vitro differentiation into functional hPSC-derived pancreatic beta cells for diabetes therapy. The methods used in this review are as follows: First, we carried out a comprehensive search of peer-reviewed original and review articles using the PubMed database and Google Scholar based on a wide range of key words linking the involvement of calcium signaling in insulin secretion, hPSCs, and diabetes. Secondly, the reference section for each selected article was searched to find additional articles to further develop this review.

## 2. Calcium Signaling in Pancreatic Beta Cells (Basics)

Systemic glycemia is carefully regulated by the precise production and release of insulin by beta cells. The interplay between blood glucose and numerous other secretagogues such as neurotransmitters, hormones, and other compounds regulates this function [27,28]. As with most other cell types, Ca^2+^ has a critical function in the controlled release of insulin from beta cells.

In the postprandial state, an elevation of blood glucose is detected by beta cells, which in turn take glucose into the cell through glucose transporters (GLUT1 in humans and GLUT2 in rodents). Glycolysis is triggered as glucose is phosphorylated by glucokinase into pyruvate. Pyruvate thus formed is transported into the mitochondria and enters the tricarboxylic acid (TCA) cycle. A cascade of events in the TCA cycle enhances ATP production. This increase in the ATP/ADP ratio within the cell inhibits ATP-sensitive K+ channels, causing membrane depolarization. L-type calcium channels are opened because of this depolarization, leading to an influx of Ca^2+^ into the cell. This wave of Ca^2+^ triggers insulin exocytosis from secretory granules [29,30].

**Figure 1:**
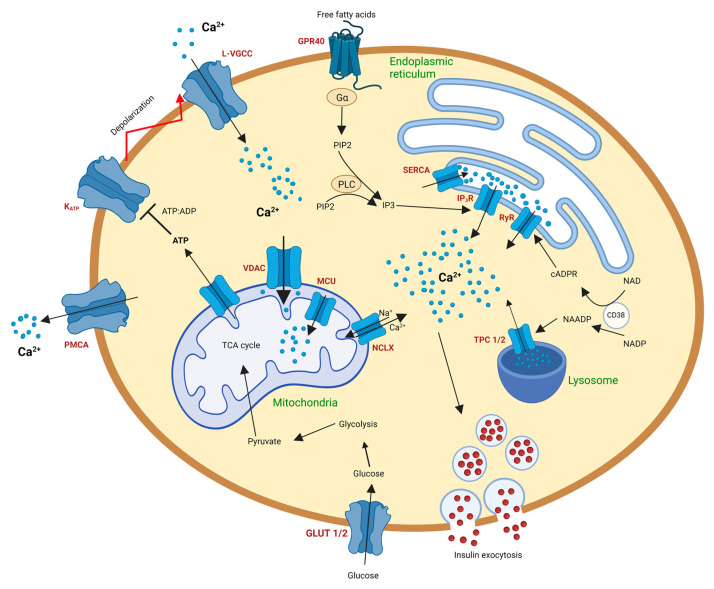
**Illustration of Ca^2+^ homeostasis in pancreatic beta cells.** Glucose enters beta cells via glucose transporters 1 or 2 (GLUT1 in humans and GLUT2 in rodents) and is converted to pyruvate by glycolysis. Pyruvate enters the mitochondria and fuels the TCA cycle, producing ATP, which shifts the ATP:ADP ratio, resulting in the closure of the ATP-sensitive potassium channel (K_ATP_). Voltage-gated Ca^2+^ channels (L-VGCC) are opened, leading to an influx of extracellular Ca^2+^ and a cascade of events resulting in insulin exocytosis. An increase in intracellular Ca^2+^ induces Ca^2+^ release from ryanodine receptors (RyR) on the endoplasmic reticulum (ER). Ca^2+^ can also be released from the ER by activation of IP3 receptors (IP3R) by IP3, which is produced from phosphatidylinositol 4,5-bisphosphate (PIP2) by the activation of phospholipase C by intracellular Ca^2+^ or fatty acid-mediated stimulation of G-protein-coupled receptors such as GPR40. Ca^2+^ reentry into the ER occurs through sarco/endoplasmic reticulum Ca^2+^-ATPase (SERCA). Ca^2+^ release from acidic organelles such as lysosomes occurs by activation of two-pore channels 1 or 2 (TPC1/TPC2) by nicotinic acid adenine dinucleotide phosphate (NAADP), which is produced by CD38 from nicotinamide adenine dinucleotide phosphate (NADP). CD38 also generates cADPR from nicotinamide adenine dinucleotide (NAD), which may elicit Ca^2+^ release from the ER through RyR. Mitochondrial Ca^2+^ entry occurs through the voltage-dependent anion channel (VDAC) and mitochondrial Ca^2+^ uniporter (MCU), and Ca^2+^ extrusion occurs through the Na^+^/Ca^2+^ exchanger (NCLX). Intracellular Ca^2+^ is extruded from the cell via plasma membrane Ca^2+^-ATPase (PMCA). Organelles are labeled in green, Ca^2+^ channels in red, Ca^2+^ is represented as blue spheres, and insulin is represented as red spheres. This illustration was created with “BioRender.com (accessed on 17 April 2023)”.

Although a major share of the intracellular Ca^2+^ is due to the influx of extracellular Ca^2+^, several intracellular organelles, including the endoplasmic reticulum (ER), nucleus, Golgi apparatus, and mitochondria, contribute to the regulation of Ca^2+^ within beta cells [31,32]. A summary of Ca^2+^ homeostasis in β-cells is shown in Figure 1.

### 2.1. Calcium Signaling in Beta Cell Endoplasmic Reticulum

The endoplasmic reticulum performs crucial functions such as protein folding and lipid synthesis, acts as a Ca^2+^ store, and effects its release [33]. Endoplasmic reticulum-mediated Ca^2+^ uptake and release are mainly dependent on inositol 1,4,5-triphosphate receptors (IP3R), ryanodine receptors (RyR), and sarco/endoplasmic reticulum Ca^2+^-ATPase (SERCA).

RyR receptors release Ca^2+^ from the ER through a positive feedback process named “calcium induced calcium release” (CICR). An increase in cytosolic Ca^2+^ triggers the opening of RyRs, which leads to an efflux of Ca^2+^ from the ER into the cytoplasm. The Ca^2+^ thus released by the ER facilitates the opening of more RyRs, hence amplifying the ER-dependent Ca^2+^ release [34]. Three isoforms of RyRs are known (RyR1, RyR2, and RyR3). RyR2 is believed to be the predominant isoform of RyR present in beta cells, although traces of RyR3 are also found [35,36]. Known activators of RyRs include cyclic AMP (cAMP), cyclic ADP ribose (cADPR), long-chain acyl CoA, and ATP [37,38]. Although the expression of RyRs in beta cells is relatively low, their high conductance capacity might be sufficient to trigger CICR in beta cells [39,40]. Additionally, drugs influencing RyR activity have been shown to have a slight effect on cytoplasmic Ca^2+^ in human beta cells without compromising GSIS activity [41]. RyR studies on islets have an additional complication wherein delta cells also express RyRs, hence distorting RyR effects on beta cells [42,43].

IP3R are IP3-sensitive receptors found on the ER membrane of many cell types, such as the cerebellum, smooth muscles, cardiac and skeletal muscle, as well as beta cells. IP3 is formed by the activation of phospholipase C, which hydrolyzes phosphatidylinositol bisphosphate (PIP2) into IP3 and diacylglycerol. IP3 is capable of mobilizing Ca^2+^ from Ca^2+^-stores and plays a significant role in the release of Ca^2+^ from the ER [44,45]. Glucose stimulation-coupled depolarizations have been indicated to promote IP3 production [46]. Studies have suggested numerous mechanisms for IP3R-mediated Ca^2+^ release from the ER. Heightened cytoplasmic Ca^2+^ levels achieved by the inhibition of SERCA have been shown to accelerate CICR due to the activation of IP3Rs [47]. CICR, independent of RyR activity, was observed when accompanied by increased levels of the second messenger cAMP in beta cells. These effects were attributed to IP3R activation [48]. In rodent islets, it was observed that ATP facilitated autocrine activation of the purinergic receptor P_2_Y_1_, which promoted ER Ca^2+^ release through IP3Rs, thereby increasing cytoplasmic Ca^2+^ levels [49]. Another mechanism of IP3R-mediated ER Ca^2+^ release is through the activation of the G-protein-coupled receptor GPR40 by free fatty acids. Fatty acid binding to GPR40 appears to activate the G_αq/11_-protein complex, leading to the production of IP3 and subsequent ER Ca^2+^ release. However, studies have also attributed the production of diacylglycerol as a determining factor in the second phase of insulin secretion [50,51,52,53]. Under ER stress, upregulation of the chaperone ERp44 rearranges IP3R and thereby reduces IP3-mediated Ca^2+^ release from the ER [54]. Genetic variations of IP3Rs have been linked as a risk factor for T1DM [55].

Ca^2+^ released from the ER is sequestered back into the ER lumen through the activity of SERCA pumps in an ATP-dependent manner. This helps maintain a resting cytosolic Ca^2+^ concentration of 25–100 nM and a high ER Ca^2+^ concentration (200–500 µM). Alternative splicing of the genes encoding SERCA generates fourteen different isoforms of SERCA. In beta cells, the predominant isoforms are SERCA2b and SERCA3 [56,57,58]. The protein phosphatase calcineurin and the kinase Protein kinase R-like ER kinase (PERK) have been shown to regulate SERCA activity in beta cells. Studies demonstrated that PERK and calcineurin work in tandem to dephosphorylate calnexin (which binds to SERCA and limits activity), thereby increasing ER Ca^2+^ reuptake [59]. The importance of the role of SERCA in normal beta cell function is well supported by the observations that SERCA2b is lost in beta cells of db/db mice and cadaveric T2DM human islets [60]. SERCA dysfunction has also been implicated in diabetic cardiomyopathy [61].

### 2.2. Calcium Signaling in the Beta Cell Lysosomes

Nicotinic acid adenine dinucleotide phosphate (NAADP) is a Ca^2+^ mobilizing messenger produced by the ADP-ribosyl cyclase CD38 by a base exchange reaction between nicotinamide adenine dinucleotide phosphate (NADP) and nicotinic acid [62]. Elevated glucose levels have been shown to increase NAADP production in beta cells [63,64]. In postprandial conditions, glucagon-like peptide 1 (GLP-1) is produced by gut L-cells and stimulates the GLP-1 receptor (GLP1R). This activates adenylyl cyclases, leading to the sequential production of NAADP [65]. Inhibition of NAADP repressed membrane depolarization and Ca^2+^ spiking, proving NAADP to be an influential player in glucose-mediated insulin release [66,67]. The consensus mode of action for NAADP is Ca^2+^ release from acidic compartments such as lysosomes and insulin granules [39]. Lysosomes are acidic organelles that contain large concentrations of Ca^2+^. Their main function is the degradation of macromolecules, but they also play a role in Ca^2+^ regulation in cells. The nanomolecular concentration of NAADP triggers lysosomal Ca^2+^ release, while micromolecular NAADP concentrations inhibit Ca^2+^ release [68]. NAADP is believed to release Ca^2+^ from the lysosome through two-pore channels (TPCs), particularly TPC2. However, recent studies have questioned TPC2 as the major NAADP receptor and suggested TPC1 as the main NAADP receptor instead. Although TPC1 and TPC2 are both known to show high sensitivity to NAADP, studies on knockout mouse models have revealed conflicting results, confounding the identity of NAADP’s primary target receptor [68,69,70,71]. Alternatively, the transient receptor potential (TRP) cation channel, particularly TRPML1, has also been suggested as a potential NAADP target receptor [72].

Adenylyl cyclases such as CD38 are also responsible for the production of another Ca^2+^ second messenger, cADPR, by the cyclization of beta-nicotinamide adenine dinucleotide. Early studies have suggested that glucose stimulation generates cADPR in pancreatic islets. Follow-up studies by the same group observed that overexpression of CD38 in transgenic mice increased glucose and ketoisocaproate-mediated insulin release, while this effect was impaired in CD38 knockout mice [73,74,75]. cADPR potentiates Ca^2+^ release from the ER through RyRs. However, evidence suggests that cADPR does not directly interact with RyR, indicating the presence of accessory proteins that act as links between cADPR and RyR. FK506-binding protein 12.6 (FKBP12.6), calmodulin, and GAPDH have been proposed as potential accessory proteins that evoke Ca^2+^ release by cADPR [76,77,78,79]. The exact mechanism tying cADPR to RyR and the subsequent release of Ca^2+^ from the ER is currently unknown. cADPR has also been shown to trigger Ca^2+^ influx and subsequent insulin secretion by activating the plasma membrane channel TRMP2 [76]. Although mounting evidence has suggested the role of cADPR as a Ca^2+^-releasing messenger, its Ca^2+^ mobilizing effects are controversial since numerous studies have failed to find an action of cADPR in beta cells [80,81,82]. It has been suggested that these inconsistencies might be due to differences in species, cell types, and experimental designs in various labs [83]. Further studies need to be performed to better understand the role of cADPR in beta cells.

### 2.3. Calcium Signaling in the Beta Cell Mitochondria

Another organelle crucial for effective GSIS is the mitochondria. The major energy-producing mechanism of the mitochondria is the TCA cycle, where the electron transport chain produces ATP. ATP generated by mitochondria promotes plasma membrane depolarization, leading to Ca^2+^ influx and insulin exocytosis. A close association between Ca^2+^ homeostasis and beta cell function has been connected to mitochondrial function [84]. Studies that manipulated mitochondrial Ca^2+^ showed impaired insulin response to high glucose challenges, thereby linking the tight regulation of Ca^2+^ and mitochondrial energy metabolism [85,86]. Three dehydrogenases of the TCA cycle have been known to be modulated by Ca^2+^, namely, pyruvate dehydrogenase, NAD^+^-isocitrate dehydrogenase, and 2-oxoglutarate dehydrogenase, and serve as crucial factors for insulin release in beta cells. The influx of Ca^2+^ into the mitochondria is believed to be essential for the stimulation of Ca^2+^-dependent dehydrogenases of the TCA cycle to control ATP production under increased glucose conditions [27,87,88,89].

Ca^2+^ entry into the mitochondria occurs in the outer mitochondrial membrane and inner mitochondrial membrane through voltage-dependent anion channels (VDACs) and mitochondrial Ca^2+^ uniporters (MCU), respectively [90,91]. Experiments with MCU knockdown in murine beta cells and the Ins-1 cell line showed a decrease in mitochondrial Ca^2+^ uptake, leading to impaired ATP production and insulin secretion. Moreover, the expression of respiratory chain complexes, mitochondrial metabolic activity, and oxygen consumption were also lowered. These studies underlined the importance of MCU-mediated Ca^2+^ regulation for sustained ATP production and metabolism-secretion coupling in beta cells [92,93]. Similar observations were made in an MCU-null mouse model, where mitochondrial Ca^2+^ uptake and GSIS were impaired [94]. The uptake and buffering of intracellular Ca^2+^ by beta cell mitochondria have been implicated in the tone and frequency of cytosolic Ca^2+^ oscillations and might perhaps contribute to insulin release pulsatility [89,95]. Mitochondrial Ca^2+^ has also been linked to the activation of the inner membrane channel, the mitochondrial permeability transition pore (PTP). PTP is believed to perform two distinct roles in beta cells, depending on its activation or inhibition. The activation of PTP is required to promote glucose-dependent insulin secretion, while its inhibition is necessary to protect against glucotoxicity and hypoxia [31,96,97,98]. Ca^2+^ extrusion from mitochondria is also key to mitochondrial and cell function. Persistent accumulation of Ca^2+^ in the matrix space can cause Ca^2+^ overload in the mitochondria, leading to the opening of PTPs and, thereby, initiating cell death signals [99]. To maintain mitochondrial Ca^2+^ homeostasis, Ca^2+^ extrusion from the mitochondria occurs via the Na^2+^/Ca^2+^ exchanger (NCLX) [100]. Studies in primary beta cells and MIN6 cell lines have shown that NCLX not only regulates mitochondrial membrane potential and calcium levels after glucose challenges, but it also controls the rate and amplitude of cytosolic Ca^2+^ responses and the rate of GSIS during the first phase of insulin secretion [101].

## 3. Role of Ca^2+^ in Beta Cell Proliferation

Insulin-positive beta cells are first seen around embryonic day 13.5 in mice and weeks 8–9 in humans. During the earlier days of fetal growth, endocrine progenitor cells differentiate into beta cells. However, during the late neonatal stages, beta cells are formed by replication [102,103,104]. There is a reduction in the rate of replication after weaning, which subsequently drops considerably in adulthood. Additionally, beta cell preservation and repair are believed to primarily occur through beta cell replication rather than adult pluripotent stem cells [104].

A study revealed that a signaling pathway linking membrane depolarization was a key factor for beta cell replication in mice. Diazoxide, a K_ATP_ channel-opening drug, reduced the replicative capability of beta cells, thereby suggesting that the requirement for the closure of K_ATP_ channels and subsequent depolarization is critical for beta cell replication. Reciprocally, when mice were injected with the K_ATP_ channel-blocking drug glyburide, beta cell proliferation was rescued. Therefore, Ca^2+^ influx appears to play an important role in beta cell replication [105]. Another study showed that the L-type voltage-gated channel agonist Bay K 8644 induced beta cell proliferation, improving our understanding of the role of Ca^2+^ and calcium channels in beta cell replication.

A known mechanism of beta cell replication is via calcineurin/nuclear factor of activated T-cells (NFAT). Calcineurin/NFAT signaling is central to the regulation of neonatal pancreatic development in mice and human islets. NFATs are activated by the serine/threonine phosphatase, calcineurin. Increasing Ca^2+^ concentrations switches on calcineurin, which in turn dephosphorylates NFAT. This leads to the translocation of NFAT into the nucleus and the transcription of multiple genes responsible for beta cell function, glucose sensing, and proliferation [106,107,108]. In human islets, the NFAT isoforms *NFATC1*, *NFATC2*, *NFATC3*, and *NFATC4* are present, with *NFATC2* and *NFATC3* being the most abundant [109]. In mouse islets, inactivation of calcineurin b1 (Cnb1) resulted in impaired cell mass and proliferation, among other dysfunctions [110]. Interestingly, inhibition of the NFAT by inhibitory kinases glycogen synthase kinase 3 beta (GSK3b) and dual tyrosine specificity kinase 1 (DYRK1A) has been shown to improve beta cell proliferation. Moreover, pharmacological interventions and genetic studies have deemed the two kinases critical for beta cell replication, and the inhibition of both has been shown to have an additive effect on the proliferation rate in primary rat beta cells [111,112]. Another study has demonstrated beta cell replication by the activation of the transcription factor c-Fos, which improves beta cell proliferation in an NKX6.1-dependent manner by the upregulation of the nuclear receptors Nr4a1, Nr4a3, and the hormone VGF. Interestingly, Nr4a1 has also been deemed critical for regulating NFATC2 in beta cell replication [113]. The calcineurin/NFAT signaling pathway may also be associated with the GLP-1 agonist receptor with respect to beta cell replication. Treatment of human islets with exendin-4, a GLP-1 receptor agonist, increases NFAT expression levels and beta cell proliferation rates. Exendin-4 has also been shown to promote beta cell proliferation by inhibiting the expression of Wnt5a [109,114]. Beta cell replication is also modulated by a Ca^2+^/calmodulin-dependent kinase (CaMK). A study observed that CaMK4 inhibition eliminated beta cell proliferation, whereas constitutively active CaMK4 expression increased beta cell proliferation via a cAMP response element binding protein (CREB) and insulin receptor substrate 2 (IRS-2) mechanism [115].

## 4. Role of Ca^2+^ in Beta Cell Survival

A multitude of studies have found a close association between Ca^2+^ handling and beta cell survival in pancreatic beta cells. High glucose enhances beta cell survival in a Ca^2+^-mediated manner. In cultured mouse islet beta cells and MIN6 insulinoma cells, a three-fold decrease in apoptosis was found in the presence of high glucose concentrations compared to low glucose concentrations. Blocking of the L-type Ca^2+^ channel with nifidepine reversed the protective effects of high glucose, suggesting the requirement of Ca^2+^ influx for beta cell survival at high glucose concentrations [116]. A recent publication studied the effects of prolonged hyperinsulinemia exposure on beta cell survival. Chronic high insulin exposure increased the intracellular Ca^2+^ in beta cells, along with an insulin dose-dependent decrease in SERCA2 expression. Prolonged high insulin exposure also triggered apoptosis in beta cells through Caspase-12 (a substrate to calcium-sensitive proteases (calpains) and a known mediator of ER stress) and correlated with an increase in intracellular Ca^2+^. This suggests that prolonged insulin exposure can disrupt ER function and induce apoptosis in beta cells. However, further studies need to be undertaken to deduce the mechanisms of these processes and the interplay between the protective and destructive effects of high glucose and insulin, respectively [117]. Ca^2+^ depletion-mediated ER stress has been associated with beta cell apoptosis in numerous studies. A study reported that Ca^2+^ depletion from the ER through dysfunctional SERCA2b, IP3Rs, and RyRs led to beta cell death and suggested a calpain-2-mediated pathway as the mechanism. The authors discussed the possibility of pharmacological modulations of the ER channels as a potential for beta cell survival [118]. Inflammatory cytokines, another potential ER stress inducer, cause ER Ca^2+^ depletion and increase cytosolic Ca^2+^ in beta cells. This led to beta cell death, marked by an increase in caspase-3 and caspase-7 activity levels. In INS-1 cells, cytokine-mediated beta cell apoptosis was inhibited by treating the cells with the calcium-modulating compounds dantrolene and sitagliptin, which restored the function of SERCA and the ER Ca^2+^ pump and decreased the levels of the pro-apoptotic protein thioredoxin-interacting protein (TXNIP) [119]. Free fatty acid-mediated beta cell apoptosis also centers around ER Ca^2+^ homeostasis. Heightened palmitate levels increase oxidative conditions, leading to Ca^2+^ depletion from the ER, which interferes with chaperones and results in misfolded proteins. This triggers a series of events where cytosolic and mitochondrial Ca^2+^ homeostasis is disrupted, thereby leading to beta cell apoptosis [120,121]. 

Calcineurin-dependent mechanisms have also been implicated in beta cell viability. Pharmacological inhibition of calcineurin is necessary to prevent organ transplant rejection and is associated with post-transplant beta cell failure. A study on human islets revealed that beta cell apoptosis was significantly increased in the presence of the calcineurin inhibitor tacrolimus. The mechanism of these effects linked calcineurin to IRS-2 and the phosphoinositide-3-kinase protein kinase B/Akt (PI3K-PKB/Akt) pathway. It was suggested that the effect of calcineurin on IRS-2 was also mediated partly through NFAT [122]. More evidence for the role of Ca^2+^ in beta cell survival was seen when CAMK4 was shown to regulate apoptosis. Constitutively active CAMK4 was shown to significantly improve beta cell survival as a reduction in caspase-3 and caspase-7 was observed. Moreover, increased apoptosis was observed in the presence of the dominant negative form of CAMK4. The authors proposed CAMK4 and its association with CREB as the primary mechanisms for improved beta cell survival. The activation of CAMK4 under intracellular Ca^2+^ elevations led to the upregulation of IRS-2 via CREB, leading to the regulation of beta cell survivability [115].

## 5. Calcium Signaling in Diabetes

As clearly highlighted in the earlier sections of this review, Ca^2+^ and its signaling pathways are critical to the proper function and survivability of pancreatic beta cells. As important as Ca^2+^ is for beta cell function, survival, and proliferation, it also plays an important role in insulin production and secretion. A plethora of studies have strongly linked Ca^2+^ dysfunction to diabetes in both human and animal models [123,124,125,126,127].

Studies have suggested the deleterious effects of long-term activation of calcium signaling pathways in beta cells. Calcineurin is a vital component of the calcineurin/NFAT pathway, which is critical to beta cell replication. However, chronic depolarization of beta cells increased calcineurin activity, which in turn reduced glucose tolerance, insulin secretion, and beta cell replication in mouse models [128]. Another publication demonstrated that mice lacking *Abcc8* (*Abcc8^−/−^* mice), a key component of the beta cell K_ATP_ channel, showed sustained membrane depolarization, leading to continued elevation of Ca^2+^ associated with dysfunctional genes involved in Ca^2+^ transport, signaling, and beta cell maintenance. Additionally, beta cells were observed to dedifferentiate and transdifferentiate into pancreatic polypeptide cells, as seen through the increased expression of the *Aldh1a3* gene (a marker for dedifferentiating beta cells). Interestingly, the expression of *Aldh1a3* was reduced by treating the cells with Ca^2+^ channel blockers, solidifying the link between sustained depolarization and beta cell functional and identity loss [129]. Another study found the presence of the stomach hormone gastrin in the islets of diabetic rodents and humans. Normally, gastrin is expressed abundantly in pancreatic embryonic development and ceases expression after birth. The authors thus observed that gastrin overexpression following hyperglycemia in the mutant mouse models and T2DM human beta cells is mediated by membrane depolarization, Ca^2+^ influx, and calcineurin signaling that could also be reversed by the normalization of glycemia [130]. A more recent publication used stem cells with knocked out *HNF1A* gene, whose deficiency impaired a network of genes crucial to calcium signaling, GSIS, and beta cell fate. These cells were then target-differentiated into beta cells. Stem cell-derived beta cells from *HNF1A* knockouts showed a reduction in the *SYT13* gene, which is responsible for calcium-regulated granule exocytosis. *HNF1A* deficiency also showed a developmental bias toward the generation of alpha cells, as an upregulation of glucagon was observed in the knockout cells. The observations from the knockout cells were comparable with those from the induced pluripotent stem cell (iPSC) line with HNF1A-MODY patient-specific mutations (R200Q) [131]. High-resolution microscopy experiments have revealed the existence of a subset of insulin granules in human beta cells and the INS-1 cell line whose exocytosis was triggered by localized Ca^2+^ influx. These pools of insulin granules were absent in T2DM donors and INS-1 cells mimicking diabetes, implicating the role of these Ca^2+^-triggered granules in the loss of rapid first-phase insulin secretion seen in T2DM patients [127]. Diabetic mice showed higher Ca_v_β_3_ (a voltage-gated Ca^2+^ channel subunit) expression, coupled with impaired calcium signaling and insulin secretion, and these effects were reduced in high-fat diet-induced diabetes in Ca_v_β_3_ deficient mice. Overexpression of Ca_v_β_3_ in human islets also showed impaired GSIS [132,133].

Glucolipotoxicity is an umbrella term to describe the detrimental effects of elevated glucose and fatty acid levels on beta cell function and survival. Glucolipotoxicity-induced beta cell failure and subsequently T2DM are associated with calcium dysregulation and ER stress, along with other non-calcium-related complications. A phenotypic screening study of compounds showed that glucolipotoxicity stress had a close association with calcium flux modulation and apoptosis. It was suggested that a Ca^2+^ overload was the main mechanism of glucolipotoxicity-mediated apoptosis and dysfunction, which was inhibited by compounds that decreased glucolipotoxicity-mediated Ca^2+^ influx into the cells [134]. The ubiquitously expressed redox regulator TXNIP has been found to be upregulated in diabetes. TXNIP overexpression led to beta cell apoptosis and glucotoxicity-induced beta cell death. It also induces inflammation activation and interleukin-1β production, which have implications for T1DM. Additionally, the lack of TXNIP promoted beta cell survival and prevented T1DM and T2DM [135,136,137,138]. The authors observed that various calcium channel blockers reduced the expression of TXNIP and deduced that the effects were due to the reduction of intracellular Ca^2+^ [139]. A phase 2 clinical study by the same group using the L-type calcium channel blocker verapamil showed improved beta cell function, lowered insulin requirements, fewer hypoglycemic events, and on-target glycemic control in patients with T1DM [140]. Free fatty acids and mediated beta cell loss via ER stress can also contribute to T2DM. Evidence for beta cell ER stress in diabetes includes enhanced expression of ER stress markers in islets of diabetic *db*/*db* mice and cultured human islets [141,142]. The free fatty acid palmitate upregulated ER stress markers and induced ER stress and apoptosis by depleting ER Ca^2+^ stores in INS-1 cells [143].

## 6. Calcium Signaling in Stem Cell-Derived Beta Cells

As discussed in the introduction section above, insulin-secreting beta cells from the pancreatic islet of Langerhans play a crucial role in the body by maintaining glucose homeostasis. It is well established that destruction or loss of beta cell mass leading to a negative impact on effective insulin secretion has emerged as the main pathogenic factor in both T1DM and T2DM [144]. Current treatment strategies, including exogenous insulin administration and islet transplantation, come with a myriad of complications, hindering them being an ideal end-point solution to treat diabetes. Recent advances in stem cell technologies have evoked great excitement and expectations for treating various disorders and dysfunctions, including diabetes. Reprogramming patient-derived somatic cells into iPSC lines followed by controlled differentiation into any cell kind makes them an attractive target for personalized treatment strategies, and since they are autologous, the risks of immune rejection are greatly reduced [145,146]. Stem cell-derived beta cells can also be used to study disease phenotypes and serve as an excellent model for drug screening studies [147,148]. Thus, beta cell replacement through iPSC technology has the potential to greatly assist in the treatment of T1DM and T2DM.

**Figure 2:**
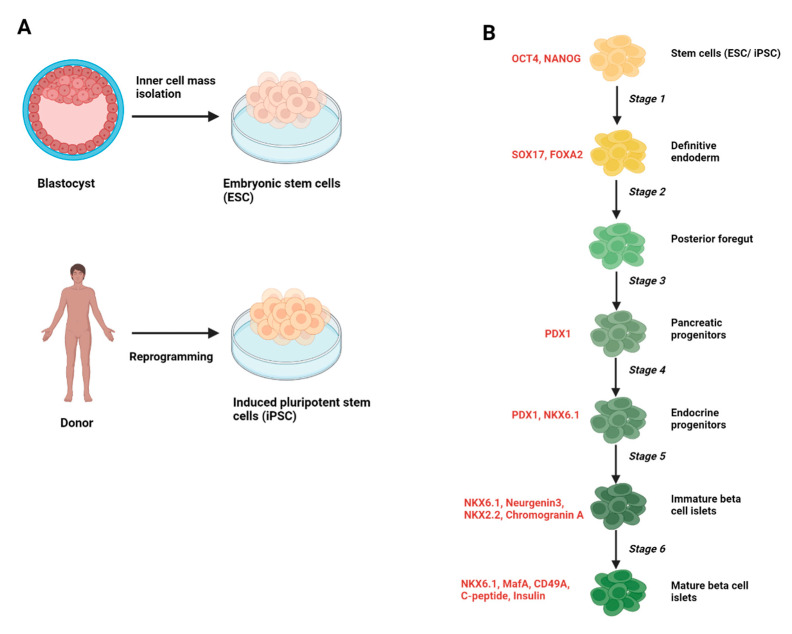
**Schematic of the generation of stem cell-derived pancreatic beta cell islets.** (**A**) Embryonic stem cells (ESC) are extracted from the inner cell mass of the blastocyst stage of the developing embryo and cultured under appropriate conditions to proliferate indefinitely. Induced pluripotent cells are generated by the direct reprogramming of somatic cells from a donor into pluripotent stem cells. (**B**) A general protocol outlining the stages of differentiation for the generation of pancreatic beta cell islets from stem cells (adapted from [149,154]). The resulting cells at each stage are labeled in black, and the quality control markers to track the efficiency (by flow cytometry or immunocytochemistry) of differentiation at each stage are labeled in red. This illustration was created with “BioRender.com (accessed on 17 April 2023)”.

Directed differentiation protocols are created from an extensive study of the developmental phases of cell types. The conversion of pluripotent stem cells into target cells is achieved by the timely addition of small molecules and growth factors in a stagewise manner that mimics the embryonic development of the desired cell type. The success of the protocol is usually determined by the extent to which these cells mirror their in vivo counterparts with respect to functional maturity and efficiency. Indeed, over the past decade, these strategies have been applied to differentiate human iPSCs and ESCs into pancreatic beta cells and islets. Pioneering work from the Melton laboratory at Harvard University has paved the way for protocols that generate beta cells with moderate efficiency. However, multiple labs have published protocols with more optimized culture conditions for differentiation media and small molecules, with the resulting protocols applicable to multiple iPSC cell lines for improved differentiation efficiency and beta cell functionality [149,150,151]. Transplantation of such generated islet-like clusters into mice has also been shown to promote their maturation and functionality [152]. A general outline for pancreatic beta cell differentiation protocols is presented in Figure 2. A common pitfall for these differentiation strategies was that the resultant beta cells showed impaired GSIS functionality compared to human islets. The GSIS response in cadaveric islets was five times higher than that observed in stem cell-derived beta cells [153].

Beta cells facilitate GSIS through increased cytosolic Ca^2+^ concentration, indicating that increased Ca^2+^ influx is a cellular function required for GSIS. Since evidence has highlighted that Ca^2+^ dysregulation in beta cells has a key role in diabetes, it is possible that functionally defective Ca^2+^ dynamics play a key role in the generation of immature and non-functionally differentiated hPSC-derived beta cells in vitro. A study used the human ESC (hESC) cell line H1 to create a seven-stage protocol to generate beta cells. The cells thus obtained expressed key markers of mature beta cells and displayed GSIS capabilities. However, as compared to adult beta cells, the differentiated cells showed reduced amplitudes and a slower time to peak of the Ca^2+^ signals. Sustained Ca^2+^ transients were observed even after the glucose stimulation ceased, a marked difference from mature beta cells. Similar effects were seen with respect to insulin release on glucose challenges, in which insulin secretion did not return to pretreatment levels and KCl-mediated insulin release was blunted. Direct depolarization with high KCl concentrations revealed the presence of voltage-gated Ca^2+^ channels and normal Ca^2+^ efflux pumps, thereby suggesting that the generated beta-like cells had functional immaturity compared to adult beta cells [150]. Another study performed a genome-wide transcriptional analysis of enriched insulin-expressing cells derived from multiple human iPSCs, human fetal pancreata, and adult human islets and drew the conclusion that iPSC-derived insulin-expressing cells resemble human fetal beta cells more than adult beta cells [26]. The authors observed that iPSC-derived insulin-positive cells had similar elevated basal glucose secretions and fared poorly under higher glucose challenges as compared to adult beta cells. The transcription factor pancreatic duodenal homeobox 1 (PDX1), which plays critical roles in early pancreas formation and several important aspects of beta cell function, is also indispensable in the maintenance of ER health [155,156,157]. A lack of the PDX1 gene altered the expression of SERCA2b, the main isoform of SERCA in beta cells that pumps Ca^2+^ into the ER, thereby impairing ER function and thereby Ca^2+^ homeostasis in beta cells [158]. A three-fold lower expression of PDX1 was found in iPSC-derived insulin-positive cells compared to adult beta cells [26]. MafA, a gene exclusively expressed in adult beta cells that promotes beta cell maturation and function, is known to be regulated by calcium signaling pathways [159,160,161]. MafA overexpression has been shown to induce GSIS in immature rat beta cells [162]. A recent publication has linked MafA regulation with Ca_v_ subunit gamma 4 (Cavγ4) in a CaMKII-mediated manner in controlling beta cell glucose homeostasis [162]. Insulin-expressing iPSC-derived cells were observed to have much lower expression of MafA compared with adult beta cells [26]. Another transcription factor that regulates beta cell mass and function is Pax4, which has also been implicated in T2DM [163]. Studies have shown that Pax4 overexpression causes increased beta cell proliferation combined with a transactivation of the antiapoptotic gene Bcl-xL. Increased Bcl-xL activity altered mitochondrial Ca^2+^ levels and ATP production, leading to impaired GSIS in islets [164]. Interestingly, Pax4 expression was significantly increased in iPSC-derived insulin-positive cells compared to adult beta cells [26]. The altered expression levels of key transcription factors such as the ones described above might impair Ca^2+^ handling in the differentiating cells and might explain the difficulty in generating beta cells functionally similar to adult human beta cells. One cannot assume that impaired calcium signaling is the major contributor to the formation of immature insulin-producing cells from iPSCs. Impaired expression of genes responsible for potassium channel regulation and mitochondrial function was also found in immature iPSC-derived beta-like cells [19,26]. Perhaps the differentiation strategies are missing key ingredients to fully activate various important genes and transcription factors across different organelles to efficiently differentiate iPSCs into functional beta cells compared to their in vivo counterparts.

Nevertheless, more recent publications have generated beta cells with comparable functionality to human islets by incorporating enrichment and reaggregation of late-stage differentiated beta-like cells for better GSIS functionality [165,166]. Cells expressing C-peptide were isolated using fluorescence-activated cell sorting (FACS), and a week-long differentiation protocol was followed after reaggregation of these cells. The aggregated enriched beta cell clusters (eBCs) contained mainly C-peptide-positive cells, although a minute population of cells expressing C-peptide, glucagon, and somatostatin were also seen. These eBCs were studied to compare their properties to those of human islets and pre-aggregation or non-enriched yet aggregated beta-like immature cells. In the presence of high glucose, the pre-aggregation cells showed a slow rise in calcium flux, which was unresponsive to lower glucose concentrations, an observation consistent with other reported beta-like cells [150]. The eBCs, however, showed a significant increase and lowering of calcium flux dependent on the concentration of the glucose challenge, as seen in human islets, suggesting the proper activation of voltage-gated Ca^2+^ channels in the eBCs. Transcriptome analysis comparing non-enriched clusters and eBCs showed an upregulation of the SCGN gene, which is responsible for calcium sensing. Moreover, genes upregulated in neonatal mouse and human fetal beta cells were seen to be upregulated in the immature beta cells, providing further evidence that immature iPSC-derived beta cells resemble fetal cells more than adult beta cells [166].

Taken together, these studies that compare the differences between iPSC-derived immature beta-like cells and iPSC-derived beta cells functionally closer to human beta cells show the importance of appropriate Ca^2+^ handling in the maturation of beta cells during their development phase in in vitro conditions, suggesting that the calcium signaling pathway is an important puzzle piece in the generation of iPSC-derived pancreatic beta-like cells. Careful considerations need to be made in the differentiation protocols to include small molecules and growth factors in the differentiation media that target the calcium signaling pathways and their components in a timely manner to generate fully functional beta cells in vitro. Current strategies have been built upon accumulating knowledge over the past decade to produce functional beta cells, and more research needs to be performed to generate high-quality iPSC-derived beta cells that resemble human beta cells.

An important tool to better understand diabetes and its progression is the integration of gene editing technology (CRISPR/Cas9) with stem cell technology. Several studies have reported the use of CRISPR/Cas9 to create stem cell-based disease models of monogenetic diabetes, MODY, and neonatal diabetes, among others, to better understand the mechanisms of disease progression [167,168,169,170]. One such study highlighted the effect of mutations in the *HNF1A* gene on the onset of MODY. CRISPR/Cas9 was used to generate *HNF1A* mutations in the hESC Mel1, which was subsequently differentiated into beta cells. The resulting beta cells were observed to have impaired GSIS and intracellular calcium levels [131]. Moreover, studies have also used CRISPR/Cas9 to correct mutated variants in disease models. Different groups have used CRISPR/Cas9 techniques to correct mutations in patient-specific iPSC lines. These iPSCs were then targeted and differentiated into beta cells [169,171]. A study used CRISPR/Cas9 in a patient-specific iPSC to correct a mutation responsible for the inactivation of the sulfonylurea receptor 1 subunit of the K_ATP_ channel in congenital hyperinsulinism. These cells were target-differentiated into beta cells and used as controls to study the effect of the mutation on beta cell function [169]. Another study used CRISPR/Cas9 to correct a diabetes-causing pathogenic variant in Wolfram syndrome 1 (*WFS1*) in IPSCs generated from a patient with Wolfram syndrome. The IPSCs were differentiated into beta cells, and it was observed that the corrected *WFS1* IPSC-derived beta cells showed strong dynamic insulin secretion and reversed streptozocin-induced diabetes following transplantation into mice [167]. Thus, CRISPR/Cas9 and its incorporation into the stem cell field promise an exciting future to better understand various diabetes disease models and device approaches to improve beta cell function and survivability. This also opens new avenues to better study the role of calcium signaling in these disease models and help formulate novel treatment strategies.

## 7. Conclusions

In this review, we emphasize the role of Ca^2+^ in the functioning of pancreatic beta cells. The role of several Ca^2+^-handling organelles have been discussed through recent studies, which highlight that the disruption of key Ca^2+^ pathways could lead to beta-cell dysfunction and thereby play a role in the onset of diabetes. We examined the key advances made in the field of stem cell technology in the study of diabetes. A comparative study of Ca^2+^ handling characteristics of stem cell-derived beta-like cells and their adult human counterparts was performed, showcasing the pitfalls of the current differentiation protocols and underlining key research strategies targeting Ca^2+^ dynamics to improve the identity and functionality of directed stem cell differentiation. Massive strides are being made to achieve the goal of generating functional beta-like cells from stem cells. Advances in the efficiency of iPSC differentiation protocols can close the gaps in the structural and functional differences between stem cell-derived pancreatic and human islets. This opens the door to fully using stem cell-derived beta cells for drug screening due to their enhanced kinetics, and the mechanisms of beta cell failure can also be studied, paving the way to greatly improve our knowledge of the intricate aspects of diabetes and its treatment, including transplantation in patients with diabetes [165,166,172].

## Data Availability

No data was used in this manuscript.
